# Distinct microbial communities in an ascidian–crustacean symbiosis

**DOI:** 10.1111/1758-2229.13242

**Published:** 2024-02-21

**Authors:** Brenna Hutchings, Susanna López‐Legentil, Lauren M. Stefaniak, Marie Nydam, Patrick M. Erwin

**Affiliations:** ^1^ Department of Biology & Marine Biology, and Center for Marine Science University of North Carolina Wilmington Wilmington North Carolina USA; ^2^ Department of Marine Science Coastal Carolina University Conway South Carolina USA; ^3^ Department of Biology SOKA University of America Aliso Viejo California USA

## Abstract

Ascidians are marine invertebrates known to occasionally host symbiotic crustaceans. Although the microbiomes of both ascidians and free‐living crustaceans have been characterized, there is no documentation of microbial communities in an ascidian–crustacean symbiosis. Samples of the solitary ascidian *Ascidia sydneiensis* and ambient seawater were collected in Belize. Four symbiotic amphipod crustaceans were retrieved from the branchial sac of the animal, and their microbiomes were compared with those from their ascidian host (tunic and branchial sac compartments) and seawater. Microbiome richness and diversity differed significantly between sample types, with amphipod microbiomes exhibiting significantly lower diversity than tunic and ambient seawater samples. Microbiome composition also differed significantly between sample types and among all pairwise comparisons, except for branchial sac and amphipod microbiomes. Differential operational taxonomic unit (OTU) analyses revealed that only 3 out of 2553 OTUs had significantly different relative abundances in amphipods compared with ascidian branchial sacs, whereas 72 OTUs differed between amphipod and tunic and 315 between amphipod and seawater samples. Thus, different body compartments of *A*. *sydneiensis* hosted distinct microbiomes, and symbiotic amphipods contained microbiomes resembling the region they inhabit (i.e., the branchial sac), suggesting that environmental filtering and co‐evolutionary processes are determinants of microbiome composition within ascidian–crustacean symbioses.

## Graphical abstract

We discovered *Amphipoda* sp. symbionts within the branchial sac of the Belizean ascidian *A*. *sydneiensis* and characterized the microbiomes of this ascidian–crustacean symbiosis. Distinct microbiomes were detected in different body compartments of the host ascidian (tunic and branchial sac), with symbiotic amphipods harbouring microbiomes resembling the region they inhabit (the branchial sac). These results suggest that environmental filtering in a shared habitat and co‐evolutionary processes may determine microbiome composition within ascidian–crustacean symbioses.

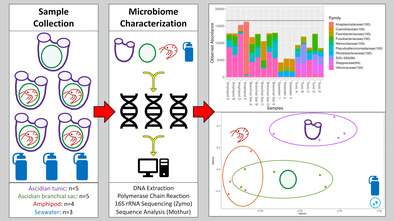

## INTRODUCTION

Ascidians (Chordata: Tunicata), also known as sea squirts, are filter‐feeding marine invertebrates found in benthic habitats across the globe, in both natural and artificial habitats. The larval stage of ascidians is short‐lived, persisting for only a few hours to days (Cloney, 1982), before larvae settle often within metres of their sessile parents (Lambert, 2005). As adults, ascidians are protected by an outer covering of cellulose called the tunic and filter ambient seawater using a branchial sac, also known as the pharyngeal gills or pharynx. These two body compartments are documented sites of symbioses with a variety of crustaceans, the majority of which belong to the phylum Arthropoda, subphylum Crustacea and classes Malacostraca (shrimp, crabs, amphipods, isopods) and Maxillopoda (copepods; San Vicente & Monniot, 2014).

Ascidians can provide a sheltered site for crustacean mating and offspring development (Ambrosio & Baeza, 2016; Ambrosio & Brooks, 2011) and protection against predation (Thiel, 2000; Thomas & Klebba, 2007). Commensal crustaceans can also take advantage of the filter‐feeding activity of their hosts to obtain food (Thomas & Klebba, 2007). In other cases, the ascidian can become infected by parasitic copepods that feed on its cellulose tunic (Hirose, 2000), suppressing the sexual reproduction of the ascidian host (Hirose et al., 2005). Multiple species and individuals of crustaceans can be present in an ascidian host simultaneously (Cruz‐Rivera et al., 2022; Kim et al., 2016), and residence in hosts is reportedly seasonal (Thiel, 2000) and dependent on the host's physiological state (White & Reimer, 2012).

Ascidians are also known to host a diverse range of microorganisms, including fungi (López‐Legentil, Erwin, et al., 2015; Matos & Antunes, 2021; Menezes et al., 2010), bacteria (Blasiak et al., 2014; Casso et al., 2020; Dror et al., 2019; Erwin et al., 2013; Erwin et al., 2014; Evans et al., 2017, 2018; Goddard‐Dwyer et al., 2021; Hirose, Hirabayashi, et al., 2006; Hirose, Hirose, et al., 2006; López‐Legentil et al., 2011) and archaea (Dror et al., 2019; Erwin et al., 2014; Evans et al., 2017; Goddard‐Dwyer et al., 2021). These microbial communities are highly host‐specific (Cahill et al., 2016; Erwin et al., 2014; Evans et al., 2017) and generally remain stable independently of geographic location (Dishaw et al., 2014; López‐Legentil et al., 2023) or time (López‐Legentil, Turon, et al., 2015). Other ascidian species can display some degree of microbiome variability in response to seasonality (Wei et al., 2020), habitat (Erwin et al., 2013; Evans et al., 2018) or the host's physiological state (López‐Legentil et al., 2016). Although most studies to date have described the microbial communities inhabiting the tunic, a few studies have also characterized gut microbiomes (Dishaw et al., 2014; Galià‐Camps et al., 2023; Wei et al., 2020) and the microbes in the branchial sac (Galià‐Camps et al., 2023; Schreiber et al., 2016). Finally, a recent study by Galià‐Camps et al. (2023) showed that within a single ascidian species, distinct microbial communities exist in the tunic, gut and branchial sac.

There is comparatively less data available on the microbiomes of crustaceans, particularly amphipods. In beach‐dwelling amphipods, microbial diversity differs significantly between members of the same species (Mengoni et al., 2013), whereas in hadal amphipods, different species harbour similar microbial communities, with slight interspecific variation due to genetics, food and environmental parameters (Chan et al., 2021). Characterization of the microbiome of symbiotic crustaceans compared with that of their marine hosts is limited to only a few studies. Examples include the microbiome of gall‐inducing copepods being species‐specific and varying depending on coral host species (Shelyakin et al., 2018) and obligate blood‐feeding crustaceans having lower microbial diversity compared with host fish (Goffredi et al., 2023). No studies to date have investigated the microbiome of amphipods associated with ascidians or the influence of resident site within the host on crustacean microbiomes, making it unclear how microbial taxa may be acquired between a host and symbiont or what functions these microbes may be providing to the symbiotic complex.

In this study, we characterized the microbial communities associated with the tunic and branchial sac of the solitary ascidian *Ascidia sydneiensis* Stimpson, 1855, the crustacean symbiont found within the branchial sac, and ambient seawater. We hypothesized that the microbiome of the amphipod would exhibit greater similarity to the microbiome of the ascidian branchial sac than that of the tunic or seawater since this was the site of residence within the host. To our knowledge, this study is the first to describe the microbial community of a symbiotic amphipod and thus will significantly advance our understanding of marine invertebrate–crustacean associations.

## EXPERIMENTAL PROCEDURES

### Sample collection, ascidian and crustacean identification

**FIGURE 1:**
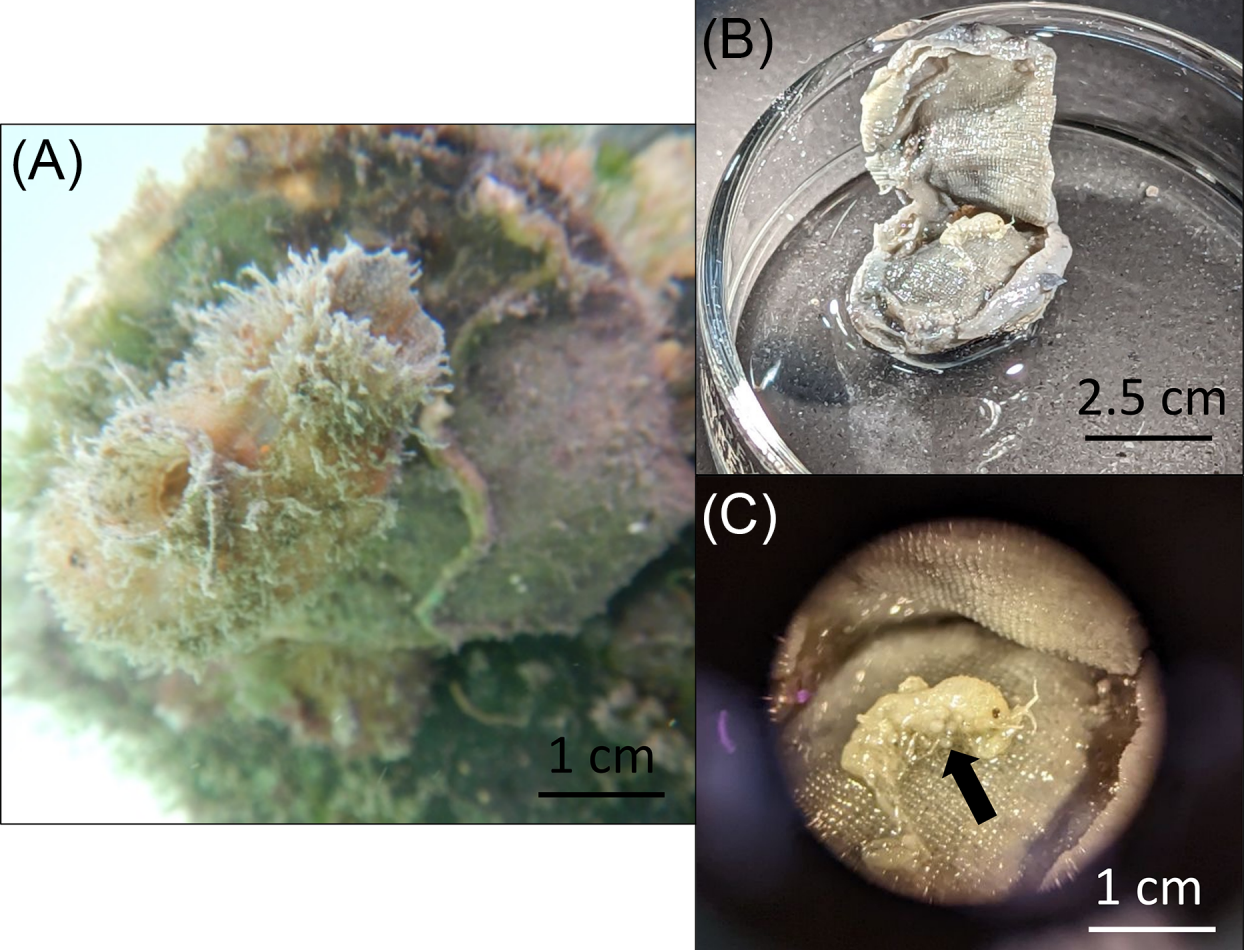
Photographs of *Ascidia sydneiensis* sampled from Thunderbirds Marina, Belize in situ (A), *Amphipoda* sp. inhabiting the branchial sac of *A*. *sydneiensis* following dissection (B) and *Amphipoda* sp. carrying eggs (arrow) while inhabiting the branchial sac (C).

Eight individuals of the solitary ascidian *A*. *sydneiensis* (Figure 1A) and three replicates of ambient seawater were collected from <1 m depth in July 2022 from Thunderbirds Marina, Placencia, Belize (16°32.587′ N, 088°21.934′ W). At the time of sampling, salinity was 30 ppt, and temperature was 30.4°C. The ascidians were immediately placed in Ziploc® bags filled with seawater and menthol crystals for at least 2 h to relax the zooids for later morphological identification. Once the animals were relaxed, three specimens were fixed in buffered formalin for taxonomic identification, and five were preserved in 95% ethanol for a few days, rinsed several times with 95% ethanol, and finally stored in 100% ethanol at −20°C for microbiome characterization. For each seawater replicate, 500 mL of ambient seawater was filtered on a 0.2‐μm filter using a Nalgene® vacuum filtration system on site, and the filter was stored in RNAlater® at −20°C.

Ascidians were identified using relevant literature (Bonnet & Rocha, 2011; Monniot, 1983; Van Name, 1945) and when dissected in the lab, individuals of *Amphipoda* sp. (Figure 1B,C) were found in the branchial sac of four *A*. *sydneiensis* replicates preserved in ethanol. Each amphipod was carefully separated from the branchial sac of the host and stored in 100% ethanol at −20°C until processed for barcoding (Text S1) using the universal primers LCO1490/HCO2198 (Folmer et al., 1994). The resulting sequence of *Amphipoda* sp. for the cytochrome oxidase I (COI) gene shared 100% identity with an *Amphipoda* sp. collected from oyster reefs in Indian River Lagoon, Fort Pierce, Florida, USA (accession number MH825923.1). Sequences obtained in this study have been deposited in GenBank® (accession numbers OR607661‐OR607662, Table S1).

The preservation of whole organisms and subsequent dissection of the tunic, branchial sac and amphipod tissue introduces the possibility of microbial exchange among body regions post‐collection. However, such homogenization was not observed herein or in previous work employing a similar methodology (e.g. Galià‐Camps et al., 2023). Indeed, we would expect to see a similar microbiome composition for all sample types if this was the case, yet we see a clear differentiation of tunic microbiomes versus branchial sac and amphipod (see below). Furthermore, similarly specialized microbiomes were detected in different ascidian tissues despite bulk preservation (Galià‐Camps et al., 2023). These results also match theoretical predictions, as ethanol preservation generally dehydrates and contracts tissue, resulting in tighter junctions and limited cellular exchange between body regions.

### Microbial symbiont processing

DNA extractions of the tunic and branchial sac of each ascidian replicate, the whole body of the amphipod crustacean and the 0.2‐μm filters obtained from the seawater samples were performed using the DNeasy® Blood and Tissue Kit (QIAGEN). Gel electrophoresis observations and DNA quantification using a NanoDrop® One Spectrophotometer were conducted to verify the quantity and quality of DNA extractions. To verify the polymerase chain reaction (PCR) viability of DNA extractions, the v4 region of the 16S rRNA gene was amplified using the primers 515f and 806r (Caporaso et al., 2011). Each PCR reaction consisted of 0.5 μL of the forward and the reverse primers, 11 μL of PCR water, 12.5 μL of MyTaq HS Red Mix and 0.5 μL of DNA. The PCR process was conducted on an Eppendorf Mastercycler Nexus X2 under the following conditions: 95°C for 2 min; 35 cycles of 95°C for 15 s, 50°C for 15 s, 72°C for 20 s; 72°C for 2 min; and a holding temperature of 10°C.

Aliquots (50 μL) of each DNA extraction were subsequently sent to Zymo Research Corporation (Irvine, California, USA) for next‐generation sequencing (Illumina MiSeq PE amplicon) of the v4 region of the 16S rRNA gene. Resequencing of select samples confirmed data reproducibility (Text S2; Table S2; Figure S1A,B). Sequences were processed in the Mothur software package (Schloss et al., 2009) following the pipeline described in Erwin et al. (2017). Briefly, sequences were aligned to the SILVA taxonomy database (v132.v4), filtered following the removal of chimeric and non‐target sequences and grouped into operational taxonomic units (OTUs) based on 97% sequence similarity (Table S3). Rare OTUs (≤10 counts) were removed as done by Goddard‐Dwyer et al. (2021), and sequence data were subsampled to the lowest sampling depth across all samples (*n* = 16,572). Microbial sequences obtained in this study have been deposited in NCBI SRA (National Center for Biotechnology Information Sequence Read Archive; accession ID: PRJNA1023864).

### Alpha‐diversity analysis

The alpha‐diversity metrics (OTU richness, Pielou's evenness and Shannon's H′ diversity) were calculated for each sample in RStudio (v.4.2.3) and compared statistically across sample types (*A*. *sydneiensis* tunic, *A*. *sydneiensis* branchial sac, *Amphipoda* sp. and seawater) using the non‐parametric Kruskal–Wallis rank sum test (package ‘stats’). Subsequent pairwise analyses were conducted using the Dunn test (package ‘fishR‐Core‐Team/FSA’ from GitHub) with Holm‐adjusted *p*‐values. Results were visualized in boxplots and rarefaction curves (packages ‘ggplot2’ and ‘vegan’).

### Beta‐diversity analysis

An non‐metric multidimensional scaling (nMDS) plot based on Bray–Curtis dissimilarity of the relative abundance of OTUs was calculated in RStudio (v.4.2.3) using the metaMDS command (packages ‘vegan’ and ‘ggplot2’) and compared statistically across sample types (*A*. *sydneiensis* tunic, *A*. *sydneiensis* branchial sac, *Amphipoda* sp. and seawater) using permutational analyses of variances (PERMANOVAs). Main PERMANOVA tests were performed using the command adonis (package ‘vegan’), and subsequent pairwise analyses were performed using the package ‘pmartinezarbizu/pairwiseAdonis/pairwiseAdonis’ from GitHub with false discovery rate (FDR)‐adjusted *p*‐values.

### Differential OTU analysis

The number and relative abundance of significantly different (FDR‐adjusted *p*‐value less than 0.05) microbial taxa between sample type pairs was determined at the OTU, genus, family, order, class and phylum levels using MicrobiomeAnalyst (Lu et al., 2023). The sequences of OTUs of interest were compared with the GenBank® database using NCBI BLASTn (Table S4).

## RESULTS

### Microbial symbiont processing

**FIGURE 2:**
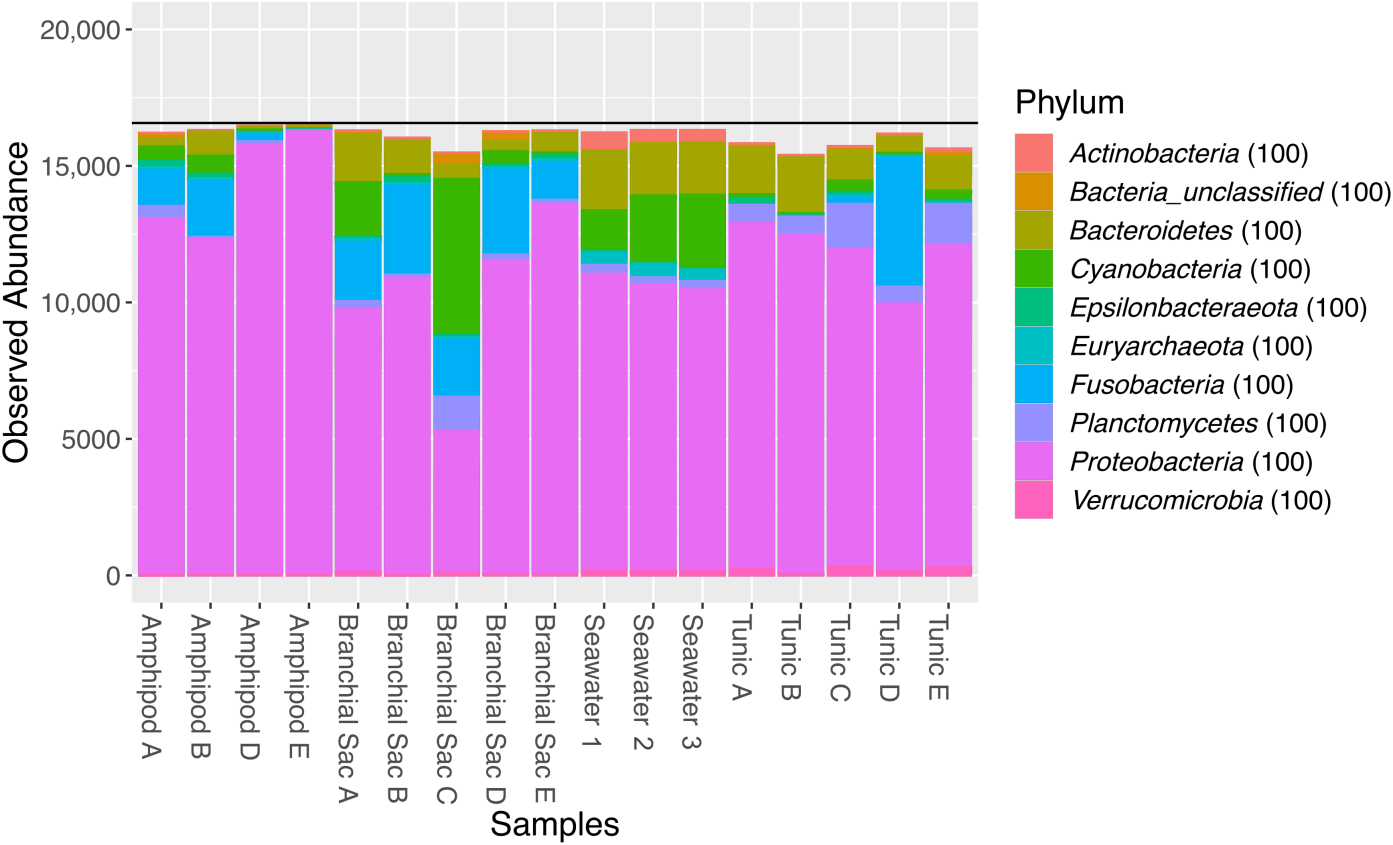
Phylum‐level composition of microbial communities in *Amphipoda* sp., *Ascidia sydneiensis* branchial sac, ambient seawater and *A*. *sydneiensis* tunic. The abundance of the 10 most common phyla is shown. The horizontal black line (*y* = 16,572) represents the sequencing depth of all samples. Letters (A–E) indicate replicate individuals of *A*. *sydneiensis*. Numbers (1–3) indicate replicates of ambient seawater. Updated Phylum names (Oren et al., 2023): Actinobacteria = Actinomycetota, Bacteroidetes = Bacteroidota, Fusobacteria = Fusobacteriota, Proteobacteria = Pseudomonadota, Verrucomicrobia = Verrucomicrobiota.

**FIGURE 3:**
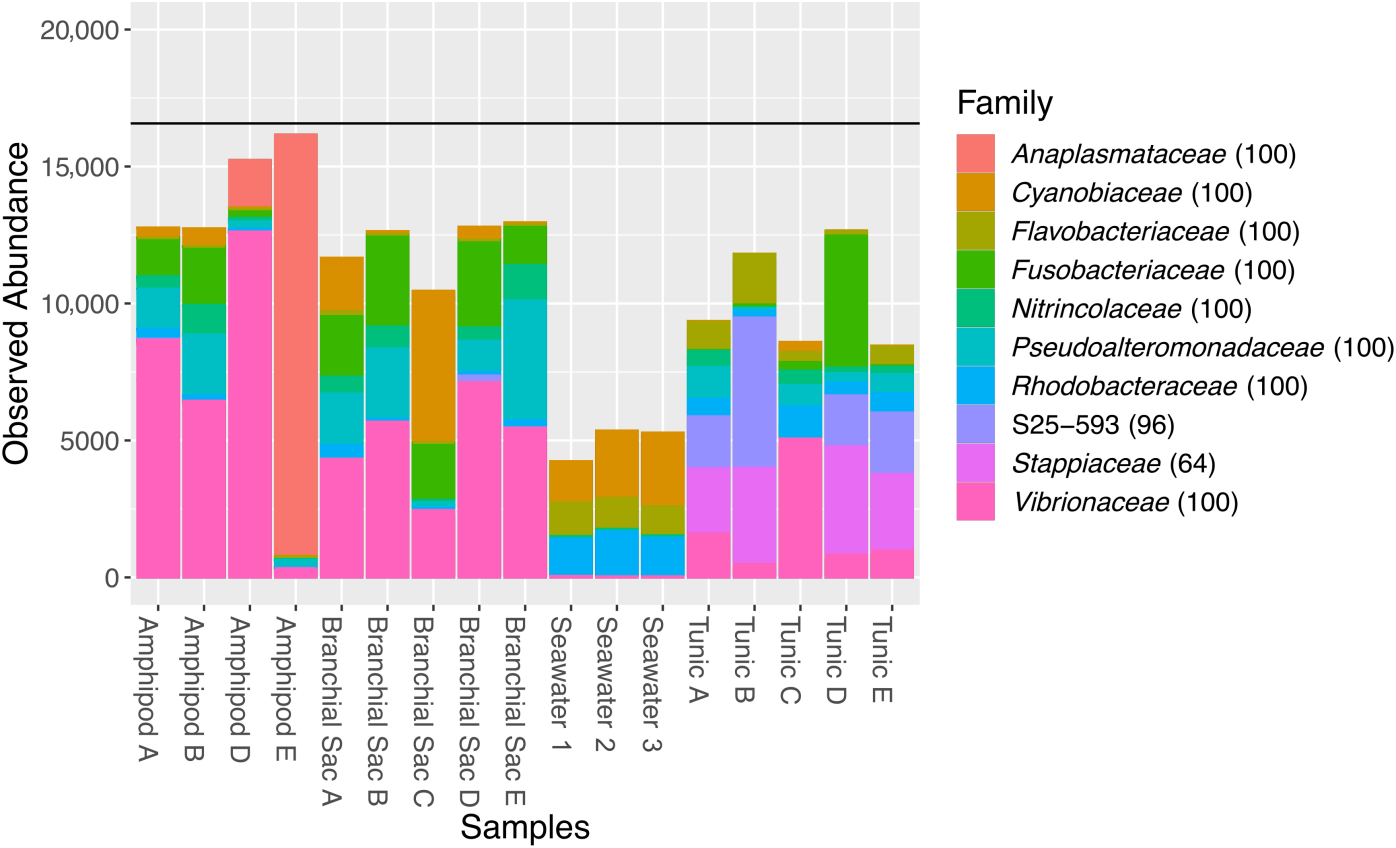
Family‐level composition of microbial communities in *Amphipoda* sp., *Ascidia sydneiensis* branchial sac, ambient seawater and *A*. *sydneiensis* tunic. The abundance of the 10 most common families is shown. The horizontal black line (*y* = 16,572) represents the sequencing depth of all samples. Letters (A–E) indicate replicate individuals of *A*. *sydneiensis*. Numbers (1–3) indicate replicates of ambient seawater.

A total of 5,488,760 raw sequences were generated from the five tunic replicates, five branchial sac replicates, four amphipod crustacean replicates and three ambient seawater replicates. These sequences clustered into 4993 OTUs. Species richness was nearing asymptotic levels in a rarefaction curve for all sample types (Figure S2A–D), indicating that microbial communities were well‐sampled. Almost all OTUs belonged to domain Bacteria, with only 0.92% of OTUs classified as Archaea (Figure S3). The most abundant phylum across all samples was *Pseudomonadota* (previously *Proteobacteria*; Figure 2), whereas *Gammaproteobacteria* and *Alphaproteobacteria* were the most abundant classes (Figure S4). Order‐level taxonomic composition was more variable (Figure S5), with *Vibrionales*, *Rickettsiales*, *Fusobacteriales* and *Alteromonadales* being the dominant taxa in the *Amphipoda* sp., ascidian branchial sac and ascidian tunic. The tunic was also dominated by *Rhizobiales* and, in the branchial sac and seawater samples, *Synechococcales* was prevalent. At the family level, *Vibrionaceae* was the most abundant for the majority of amphipod and branchial sac samples, with *Anaplasmataceae* being extremely abundant in one of the amphipod replicates. *Fusobacteriaceae* and *Pseudoalteromonadaceae* were fairly abundant in all ascidian and amphipod samples, whereas *Cyanobiaceae* was the most abundant family in seawater samples. Tunic samples were also dominated by *Stappiaceae* (Figure 3). At the level of genus, *Catenococcus* was most abundant in amphipod and branchial sac samples, except for the amphipod replicate dominated by an unclassified *Anaplasmataceae*. *Propionigenium* and *Pseudoalteromonas* were present in all ascidian and amphipod samples, whereas *Synechococcus*‐CC9902 was the most abundant in seawater (Figure S6).

### Alpha diversity analysis

**FIGURE 4:**
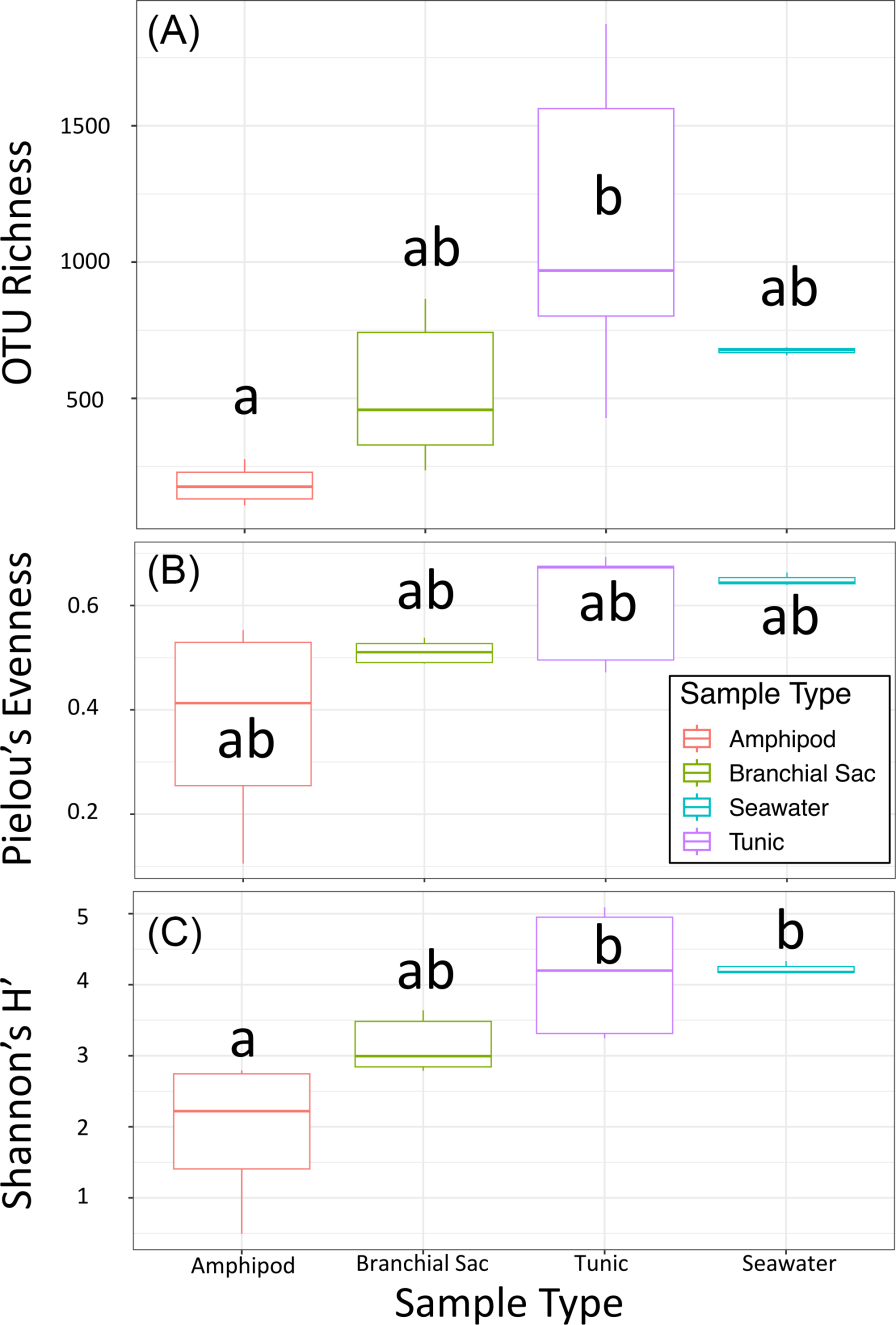
Box plots of microbial community alpha‐diversity in *Amphipoda* sp. (red), *Ascidia sydneiensis* branchial sac (green), *A*. *sydneiensis* tunic (purple) and ambient seawater (blue). Operational taxonomic unit (OTU) richness (A), Pielou's evenness (B) and Shannon's H′ diversity (C) are shown by sample type. Letters denote significant differences between sample types.

Observed OTU richness differed significantly among sample types (*χ* ^2^ = 10.402, *p* = 0.015), with amphipod microbiomes exhibiting significantly lower richness than ascidian tunic microbiomes (*p*‐adj = 0.008; Figure 4A; Table 1). There was no significant difference in evenness among sample types (*χ* ^2^ = 5.718, *p* = 0.1262; Figure 4B; Table 1). Shannon's H′ diversity significantly differed among sample types (*χ* ^2^ = 11.552, *p* = 0.009), with amphipod samples displaying significantly lower diversity than ascidian tunic (*p*‐adj = 0.018) and ambient seawater microbiomes (*p*‐adj = 0.030; Figure 4C; Table 1).

**TABLE 1:** Pairwise comparisons (Dunn tests) of microbiome richness, Pielou's evenness and Shannon's H′ diversity among sample types.

Pairwise comparison	Richness			Evenness			Diversity		
	*z*	*p*	*Holm*	*z*	*p*	*Holm*	*z*	*p*	*Holm*
Amphipod × branchial sac	−1.786	0.074	0.296	−0.443	0.658	1.000	−1.432	0.152	0.304
Amphipod × tunic	−3.203	0.001	**0.008**	−1.742	0.082	0.408	−2.967	0.003	**0.018**
Amphipod × seawater	−1.880	0.060	0.301	−1.945	0.052	0.311	−2.744	0.006	**0.030**
Branchial sac × tunic	−1.503	0.133	0.399	−1.378	0.168	0.505	−1.628	0.104	0.414
Branchial sac × seawater	−0.325	0.745	0.745	−1.627	0.104	0.415	−1.555	0.120	0.360
Tunic × seawater	−0.976	0.329	0.658	0.434	0.664	0.664	0.145	0.885	0.885
	*χ* ^2^	*p*	‐	*χ* ^2^	*p*	‐	*χ* ^2^	*p*	‐
Kruskal–Wallis	10.402	**0.015**		5.718	0.126		11.552	**0.009**	

*Note*: *Z*‐scores (*z*), *p*‐values (*p*) and Holm‐adjusted *p*‐values (*Holm*) are reported. For comparison across all samples, Kruskal–Wallis *χ* ^2^ and *p*‐values (*p*) are reported. Bold text denotes significant results.

### Beta‐diversity analysis

**FIGURE 5:**
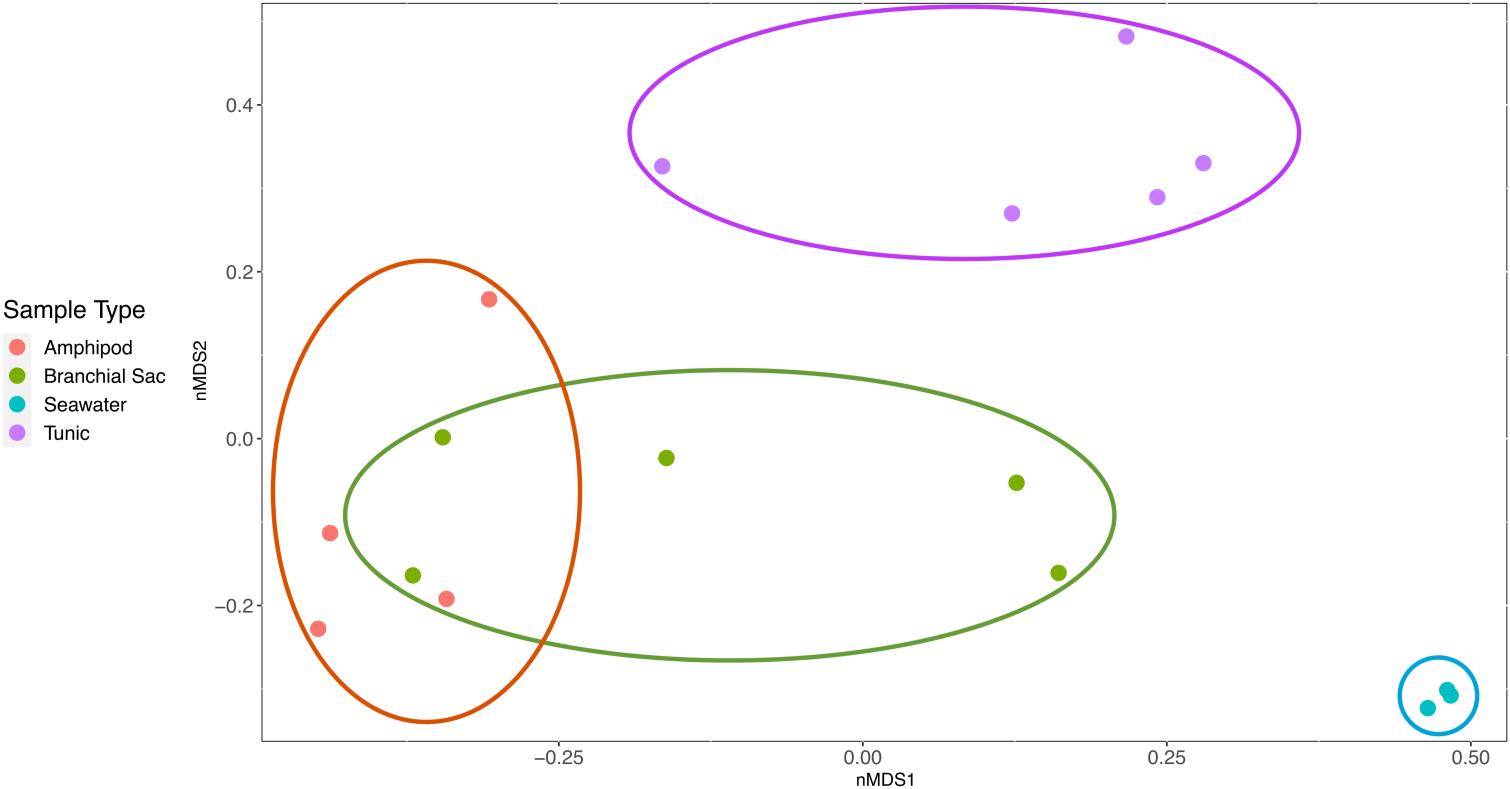
Non‐metric multidimensional scaling (nMDS) plot of microbial communities in *Amphipoda* sp. (red), *Ascidia sydneiensis* branchial sac (green), *A*. *sydneiensis* tunic (purple) and ambient seawater (blue) based on Bray–Curtis similarity of relative abundance data. Ellipses encompass all replicates within each sample type. Stress = 0.053.

Microbial community composition was significantly different among sample types (PERMANOVA, *p* = 0.001, *R* ^2^ = 0.585), and the nMDS plot (stress = 0.053) illustrated distinct microbial communities for all sample types, with more overlap in community similarity between the branchial sac and amphipod crustacean samples (Figure 5). Accordingly, significant differences in community similarity were reported for each pairwise comparison (*p* < 0.050), except for the ascidian branchial sac and amphipod samples (*p* = 0.098; Table 2).

**TABLE 2:** Pairwise comparisons of microbial community similarity (PERMANOVA) between sample types.

Pairwise comparison	*R* ^2^	*p*	FDR
Amphipod × branchial sac	0.181	0.098	0.098
Amphipod × tunic	0.338	0.019	**0.036**
Amphipod × seawater	0.607	0.036	**0.043**
Branchial sac × tunic	0.417	0.008	**0.036**
Branchial sac × seawater	0.722	0.024	**0.036**
Tunic × seawater	0.646	0.012	**0.036**
PERMANOVA	0.585	**0.001**	‐

*Note*: *R* ^2^, *p*‐values and false detection rate (FDR)‐adjusted *p*‐values are reported. For comparison across all samples, permutational analyses of variance (PERMANOVA) *R* ^2^ and *p*‐values (*p*) are reported. Bold text denotes significant results.

### Differential OTU analysis

In terms of relative abundance, at the OTU level, only 3 of 2553 OTUs (0.12%) differed significantly between the amphipod and the branchial sac microbiomes. Comparatively, 72 OTUs (2.82%) differed significantly between amphipod and tunic, 315 (12.34%) between amphipod and seawater, 103 (4.03%) between branchial sac and tunic, 324 (12.69%) between branchial sac and seawater and 504 (19.74%) between tunic and seawater (Tables 3 and 4).

**TABLE 3:** Pairwise comparisons showing the number of significantly different microbial taxa (FDR‐adjusted *p* ≤ 0.05) by taxonomic rank for each sample type pair.

	Taxonomic level					
Pairwise comparison	OTU	Genus	Family	Order	Class	Phylum
Amphipod × branchial sac	3	2	5	4	4	2
Amphipod × tunic	72	71	56	46	21	9
Amphipod × seawater	315	99	71	44	17	9
Branchial Sac × tunic	103	81	60	47	19	7
Branchial Sac × seawater	324	124	77	52	20	9
Tunic × seawater	504	163	104	70	28	15
Total taxa by level	2553	519	311	199	81	39

*Note*: The bottom row depicts the total number of taxa present at each level.

Abbreviations: FDR, false discovery rate; OTU, operational taxonomic unit.

**TABLE 4:** Identity and relative abundance (RA) of microbial taxa that differed significantly (FDR‐adjusted *p* ≤ 0.05; *p*) between the amphipod (Amph.) and ascidian branchial sac (Bran.) microbiomes.

Taxa level	Identity	Amph. RA	Bran. RA	*p* value
OTU	OTU00198 *Ferrimonas*	0.009%	0.118%	2.15 × 10^−5^
	OTU00256 *Deltaproteobacteria* (unclassified)	0.014%	0.144%	0.000
	OTU00062 SAR116 Clade	0.000%	0.014%	0.002
Genus	*Desulfobacteraceae* (unclassified)	0.003%	0.088%	0.000
	SVA0081 sediment group	0.000%	0.060%	0.002
Family	*Desulfobacteraceae*	0.003%	0.200%	8.44 × 10^−7^
	*Syntrophobacteraceae*	0.005%	0.068%	6.69 × 10^−5^
	*Pirellulaceae*	0.368%	1.715%	0.004
	*Bacteria* (unclassified)	0.382%	1.512%	0.006
	*Saccharospirillaceae*	0.002%	0.173%	0.007
Order	*Syntrophobacterales*	0.005%	0.075%	5.25 × 10^−5^
	*Methanomicrobiales*	0.000%	0.072%	0.002
	*Pirellulales*	0.368%	1.715%	0.004
	*Bacteria* (unclassified)	0.382%	1.512%	0.006
Class	*Methanomicrobia*	0.000%	0.119%	1.35 × 10^−6^
	*Planctomycetacia*	0.453%	1.840%	0.005
	*Bacteria* (unclassified)	0.382%	1.512%	0.006
	*Acidobacteriota* (previously *Acidobacteria*) Subgroup 22	0.012%	0.030%	0.008
Phylum	*Euryarchaeota*	0.008%	0.163%	0.000
	*Bacteria* (unclassified)	0.382%	1.512%	0.006

*Note*: Identity represents the lowest taxonomic rank level.

Abbreviations: FDR, false discovery rate; OTU, operational taxonomic unit.

Similarly, the percentage of significantly differential taxa in terms of relative abundance was lower between the amphipod and branchial sac than for any other pairwise comparisons across all taxonomic levels. For genera, there were 2 of 519 (0.39%) significantly different taxa between the amphipod and branchial sac, while all other pairwise comparisons exhibited 71–163 differences (≥13.68%; Tables 3 and 4). At the family level, 5 of 311 (1.61%) taxa were significantly different between amphipod and branchial sac, whereas all other pairwise comparisons exhibited 56–104 differences (≥18.01%, Tables 3 and 4). At the order level, the amphipod and branchial sac comparison resulted in 4 of 199 (2.01%) significantly differing taxa, whereas all others had 44–70 differences (≥22.11%; Tables 3 and 4). At the class level, 4 of 81 (4.94%) taxa significantly differed between the amphipod and branchial sac, whereas 17–28 (≥20.99%) differed in all other pairwise comparisons (Tables 3 and 4). Finally, at the phylum level, 2 of 39 (5.13%) taxa significantly differed between the amphipod and the branchial sac, with the other pairwise comparisons having 7–15 differences (≥17.95%; Tables 3 and 4).

## DISCUSSION

Here, we have characterized the microbiomes associated with the branchial sac and tunic of *A*. *sydneiensis*, the whole body of a symbiotic *Amphipoda* sp. and ambient seawater, resulting in the identification of 4993 OTUs. In the ascidian, different compartments (tunic and branchial sac) hosted significantly different microbial communities. This same observation was recently documented among the tunic, branchial sac and gut compartments of the solitary ascidian *Styela plicata* (Galià‐Camps et al., 2023), suggesting that the function and physical structure of the ascidian tissues influence microbiome composition. While the tunic is a passive structure, the branchial sac serves as a filter to actively trap particulates from seawater, leading to greater incorporation of seawater microbes into the branchial sac microbiome (Galià‐Camps et al., 2023). Accordingly, the microbial community in the branchial sac of *A*. *sydneiensis* shared more similarities with the microbial seawater communities than did the tunic.

Microbial communities in the amphipod were significantly less rich than those in ascidian tunic samples and less diverse than tunic and ambient microbial seawater communities. High levels of microbial diversity have been documented in the tunics of multiple ascidian species, with microenvironments encouraging microbial diversity due to varying chemical substrates, oxygen and light availabilities across the tunic (Behrendt et al., 2012; Erwin et al., 2014). The *Amphipoda* sp. microbiome was compositionally similar to the microbiome of the ascidian branchial sac but different from those of the tunic and seawater. By holding residence within the filter‐feeding branchial sac of *A*. *sydneiensis*, the symbiotic *Amphipoda* sp. is exposed to the same ciliary‐mediated water flow, irradiation levels, oxygen availability and particulates. Ciliated surfaces like the branchial sac of ascidians promote microbial settlement in other hosts by creating unique flow dynamics (Nawroth et al., 2017) and, as previously discussed, light and oxygen variability is influential in shaping microbial communities in ascidian hosts (Behrendt et al., 2012; Erwin et al., 2014). Additionally, the ascidian branchial sac contains mucus that can support the growth of facultative microbes horizontally acquired from seawater (Schreiber et al., 2016). Thus, a complex interaction of abiotic and physiological factors in the ascidian branchial sac may select for distinct microbial communities in this host body region and in resident amphipods therein. In addition to such environmental filtering, the co‐evolution of host ascidian and symbiotic amphipod microbiomes may have resulted in a similar community structure. For example, if microbial taxa are vertically transmitted across generations of *A*. *sydneiensis* and *Amphipoda* sp., metabolic interdependence between both host species and their microbiomes may evolve, creating a unique holobiont structure (Koskella & Bergelson, 2020).

A few microbial taxa did significantly differ in terms of relative abundance between the branchial sac and the amphipod, were more abundant in the former than the latter and were related to specific metabolic pathways. For example, *Ferrimonas* is a facultative reducer of Fe(III) and has been observed in filter‐feeding sponges (Abbas & Mahmoud, 2022) and suspension‐feeding sea cucumbers (Kang et al., 2023). SAR116 is an alphaproteobacteria found globally in surface seawater that plays an important role in the sulphur cycle (Roda‐Garcia et al., 2021). Members of the family *Pirellulaceae* are known to assist their sea cucumber hosts in obtaining energy from the polysaccharides in algae (Feng et al., 2022) and contribute to the core microbiomes of coral (Kellogg et al., 2017). Family *Saccharospirillaceae* also secrete carbohydrate‐active enzymes (Leadbeater et al., 2021); thus, these two families may assist *A*. *sydneiensis* in breaking down the cyanobacteria present within the branchial sac. In addition to these bacterial taxa, the archaeal family *Methanomicrobiales* was found in low abundance in the branchial sac but was absent in amphipod samples. This obligate anaerobic archaea produces methane (Sarmiento et al., 2011), and genes involved in methane metabolism were previously documented in the microbiome of some ascidian species' tunic (Matos et al., 2020) and gut (Wei et al., 2020). Thus, our study adds the branchial sac compartment to the list of ascidian body regions with methanogenic archaea.

Although not significantly different in relative abundance among amphipod and ascidian samples, a particularly interesting bacterial family detected at an abundance ~37× higher in the branchial sac than in the amphipod was identified as *Chitinophagaceae* (Phylum *Bacteroidota*, previously *Bacteroidetes*). This family is commonly found in soils and sediments and can efficiently degrade biopolymers (Gomes et al., 2010) and remove organic pollutants such as polycyclic aromatic hydrocarbons (PAHs; Blanco‐Enríquez et al., 2018). *Chitinophagaceae* has been documented in polluted mangrove soils, particularly alongside the water‐soluble PAH naphthalene (Fiard et al., 2022), and its presence in the branchial sac of *A*. *sydneiensis* suggests that it may assist the ascidian in surviving in the likely polluted marina where it was collected.

Another bacterial family of note is *Anaplasmataceae* (Phylum *Pseudomonadota*, previously *Proteobacteria*), which was the dominant taxa within one of our amphipod replicates. A genus within *Anaplasmataceae*, *Wolbachia*, contains species that act as reproductive parasites in arthropods (Werren et al., 2008) and can infect amphipods and isopods through horizontal transmission (Cordaux et al., 2001), as well as mud crabs (Bojko et al., 2022). BLASTn results of our *Anaplasmataceae* sequence shared 94.82% identity with a strain extracted from a mud crab, indicating a potential novel genus of the family found in our amphipod sample. Since the *Anaplasmataceae* family was only highly abundant in a single individual of *Amphipoda* sp., it is possible that the individual was infected before it entered its ascidian host, with no evidence of transmission of the bacteria to *A*. *sydneiensis*.

Across all samples, *Pseudomonadota* (previously *Proteobacteria*) were the most abundant, specifically the classes *Alphaproteobacteria* and *Gammaproteobacteria*. Similar to the *Amphipoda* sp. observed here, zooplankton amphipods (De Corte et al., 2018; Shoemaker & Moisander, 2015) and talitrid amphipods (Mengoni et al., 2013) host high abundances of alphaproteobacteria, whereas hadal amphipods (Chan et al., 2021) have high numbers of gammaproteobacteria. At lower taxonomic levels, family *Vibrionaceae* (particularly *Catenococcus*) and the genera *Propionigenium* and *Pseudoalteromonas* were present across all ascidian and amphipod samples. Members of *Vibrionaceae* have been documented in hadal amphipods (Chan et al., 2021) and *Vibrio* strains have been documented in relatively high counts in ascidian tunic (Utermann et al., 2020). Members of the genus *Catenococcus* can form biofilms (Wang et al., 2022), display antimicrobial and antioxidant activity (Yoghiapiscessa et al., 2016), be pathogenic in corals (Fifer et al., 2022), oxidize thiosulfate and reduce sulphur (Sorokin et al., 1995). *Pseudoalteromonas* has been previously documented in the branchial sac of ascidians (Galià‐Camps et al., 2023; Schreiber et al., 2016), and the antifouling bacterium *P*. *tunicata* was isolated from the solitary ascidian *Ciona intestinalis* (Holmström et al., 1998). Finally, *Propionigenium* species are anaerobic bacteria that decarboxylate succinate to propionate as they grow and were first isolated from anoxic marine sediments (Schink, 1992). Thus, these shared bacterial genera in our ascidian and amphipod samples may be playing a defensive role for their hosts and facilitate nutrient access, with anaerobic bacteria likely sequestered within anoxic microhabitats formed across tissues (Behrendt et al., 2012).

Our study provides significant insight into the structural factors and putative functionality of microbial communities in ascidians and their symbiotic amphipods, yet the nature of this ascidian–crustacean symbiosis remains mostly unclear. The amphipod may benefit from the ascidian filtration activity to obtain food (Thomas & Klebba, 2007), which could result in the similar microbial communities observed herein with the branchial sac. In addition, the amphipod may rely on the ascidian for reproductive purposes (Thiel, 2000), as suggested by the presence of eggs in one of the observed samples. The effect of the amphipod on the host also needs to be documented and would require long‐term monitoring of the symbiosis and its effect on the host's biological cycle. From a microbial perspective, additional characterization of microbial communities in macrosymbionts and metagenomic approaches to elucidate the function of those microbes will help determine microbial contributions to the ecology of ascidian–crustacean symbioses and dependency between host and symbiont.

## AUTHOR CONTRIBUTIONS

**Brenna Hutchings:** Conceptualization (lead); formal analysis (lead); investigation (equal); writing – original draft (lead); writing – review and editing (lead). **Susanna López‐Legentil:** Conceptualization (supporting); formal analysis (supporting); funding acquisition (equal); investigation (equal); writing – original draft (supporting); writing – review and editing (supporting). **Lauren M. Stefaniak:** Funding acquisition (equal); investigation (equal); writing – review and editing (supporting). **Marie Nydam:** Funding acquisition (equal); investigation (equal); writing – review and editing (supporting). **Patrick M. Erwin:** Conceptualization (supporting); formal analysis (supporting); funding acquisition (equal); investigation (equal); writing – original draft (supporting); writing – review and editing (supporting).

## CONFLICT OF INTEREST STATEMENT

The authors report no conflicts of interest.

## Supporting information


**Data S1.** Supporting Information.

**Figure S1.** Visual comparisons between technical replicates sequenced for this study. OTU richness is plotted for each replicate (A), where samples collected from the same individual of *Ascidia sydneiensis* have a label starting with A–E, corresponding to the five individuals used in this study. Crustacean samples are noted with an ‘R,’ branchial sac with an ‘S,’ tunic with a ‘T,’ and seawater with a ‘W’. A code followed by a ‘1’ indicates it was the result of the first sequencing procedure, while a ‘2’ indicates the product of the second sequencing procedure. Three samples were only produced in the second sequencing procedure: BR, CT and ET (marked with a black star). An nMDS plot comparing microbiome composition among samples is shown (B), where non‐mathematically derived ellipses indicate sample type: amphipod crustaceans (red), branchial sac (green), tunic (purple) and ambient seawater (blue). A line connects each technical replicate pair.

**Figure S2.** Rarefaction curves of microbial OTU richness for *Amphipoda* sp. samples (A, red), *Ascidia sydneiensis* branchial sac (B, green), ambient seawater (C, blue) and *A*. *sydneiensis* tunic (D, purple). Letters (A–E) indicate replicate individuals of *A*. *sydneiensis*. Numbers (1–3) indicate replicates of ambient seawater.

**Figure S3.** Domain level composition of microbial communities in *Amphipoda* sp., *Ascidia sydneiensis* branchial sac, ambient seawater and *A*. *sydneiensis* tunic. Horizontal black line (*y* = 16,572) represents the sequencing depth of all samples.

**Figure S4.** Class level composition of microbial communities in *Amphipoda* sp., *Ascidia sydneiensis* branchial sac, ambient seawater and *A*. *sydneiensis* tunic. Abundance of the 10 most common classes is shown. Horizontal black line (*y* = 16,572) represents the sequencing depth of all samples.

**Figure S5.** Order‐level composition of microbial communities in *Amphipoda* sp., *Ascidia sydneiensis* branchial sac, ambient seawater and *A*. *sydneiensis* tunic. Abundance of the 10 most common orders is shown. Horizontal black line (*y* = 16,572) represents the sequencing depth of all samples.

**Figure S6.** Genus‐level composition of microbial communities in *Amphipoda* sp., *Ascidia sydneiensis* branchial sac, ambient seawater and *A*. *sydneiensis* tunic. Abundance of the 10 most common genera is shown. Horizontal black line (*y* = 16,572) represents the sequencing depth of all samples.

## Data Availability

Data files and raw microbial sequence data are available from the corresponding author. All COI sequences acquired in this study have been deposited in GenBank® (accession numbers OR607661 to OR607662) and all 16S rRNA sequences have been deposited in NCBI SRA (accession ID: PRJNA1023864).
