# Supplementary material for: Distinct microbial communities in an ascidian–crustacean symbiosis
Source: Environ Microbiol Rep. 2024 Feb 21;16(1):e13242. doi: 10.1111/1758-2229.13242 (PMC10881349; doi:10.1111/1758-2229.13242)
Supplement: Supplementary file 1 — Data S1. Supporting Information. [file EMI4-16-e13242-s004.docx]

**SUPPLEMENTARY MATERIALS**

**Distinct microbial communities in an ascidian-crustacean symbiosis**

Brenna Hutchings^1^, Susanna López-Legentil^1^, Lauren M. Stefaniak^2^, Marie Nydam^3^, Patrick M. Erwin^1^

^1^*Department of Biology & Marine Biology, and Center for Marine Science, University of North Carolina Wilmington, 5600 Marvin K. Moss Lane, Wilmington NC 28409, United States of America*

^2^*Department of Marine Science, Coastal Carolina University, 100 Chanticleer Dr. E., Conway SC 29528, United States of America*

^3^*Department of Biology, SOKA University of America, 1 University Drive, Aliso Viejo CA 92656, United States of America*

Text S1

For barcoding of the *Amphipoda* sp., the whole amphipod was separated from the ascidian host and placed in 100% ethanol at -20^o^C until processed. An Eppendorf^®^ Vacufuge^®^ centrifuge was used to evaporate any ethanol from the amphipod tissue and DNA extractions were performed with the DNeasy^®^ Blood and Tissue Kit (QIAGEN) following manufacturer’s instructions. The universal primers LCO1490/HCO2198 (Folmer et al. 1994) were used to amplify a fragment of the mitochondrial gene Cytochrome Oxidase I (COI) via PCR. Each PCR reaction consisted of 0.5µL of the forward and reverse primers, 11µL of PCR water, 12.5µL of MyTaq HS Mix, and 0.5µL of DNA. PCR reactions were conducted on an Eppendorf Mastercycler Nexus X2 as follows: 95^o^C for 1 minute; 35 cycles of 95^o^C for 15 seconds, 45^o^C for 15 seconds, 72^o^C for 10 seconds; 72^o^C for 1 minute; and a holding temperature of 4^o^C.

PCR products underwent PCR purification using the QIAquick PCR Purification Kit (QIAGEN) following manufacturer’s instructions, followed by a sequencing reaction consisting of 5.5µL PCR water, 1.0µL BigDye^TM^ Terminator v.3, 2.0µL BigDye^TM^ Buffer (x5 concentration), 0.5µL of forward or reverse primers, and 1.0µL of purified PCR product. Sequencing reactions were conducted on an Eppendorf Mastercycler Nexus X2 as follows: 96^o^C for 1 minute; 25 cycles of 50^o^C for 5 seconds, 60^o^C for 4 minutes, 96^o^C for 10 seconds; 50^o^C for 5 seconds; 60^o^C for 4 minutes; and a holding temperature of 10^o^C.

Samples were sequenced on an Applied Biosystems 3500 genetic analyzer (located at UNCW Center for Marine Science) using the BigDye™ XTerminator Purification Kit (Applied Biosystems) following manufacturer’s instructions and corresponding primers. Retrieved DNA sequences were aligned in Geneious (v. R11. Biomatters, Auckland, New Zealand) and compared to published sequences available in GenBank^®^ via BLASTn searches.

Text S2

Two aliquots for each sample were sent for sequencing as technical replicates to assess reproducibility. Technical replicates exhibited high reproducibility (Figure S1) and no significant differences in alpha-diversity (pairwise Dunn test with Holm-adjusted p-values) and beta-diversity (pairwise permutational analyses of variances (PERMANOVAs) with false discovery rate adjusted p-values) were detected across runs (Table S3). Accordingly, the second sequencing run was selected for use in the final dataset due to more complete representation of samples (Ex. Run 1= 75% of amphipod and 60% of tunic samples were successful; Run 2= 100% of amphipod and 100% of tunic samples were successful).

Supplemental References

Folmer O, Black M, Hoeh W, Lutz R, Vrijenhoek R (1994) DNA primers for amplification of mitochondrial cytochrome *c* oxidase subunit I from diverse metazoan invertebrates. Mar Biotechnol 3(5):294-299

Supplemental Tables

**Table S1** Sample code of *Amphipoda* sp. replicates, sequenced gene region, published GenBank^®^ accession numbers (Acc. num), closest BLASTn match with a published sequence (species and % identity), and origin and accession number of BLASTn sequence. Different accession numbers for a given species correspond to different haplotypes.

| Species | Code | Gene | Acc. num | BLASTn | Origin | Acc. num |
| --- | --- | --- | --- | --- | --- | --- |
| *Amphipoda* sp*.* | 25Jul22-1-2AR | COI | OR607662 | *Amphipoda* sp. FTP_3358 (100%) | Indian River Lagoon, Fort Pierce, Florida, USA | MH825923.1 |
|  | 25Jul22-1-2DR | COI | OR607661 | *Amphipoda* sp. FTP_3358 (100%) | Indian River Lagoon, Fort Pierce, Florida, USA | MH825923.1 |

**Table S2** Pairwise comparisons (Dunn tests, alpha-diversity) of microbiome richness, Pielou’s evenness, and Shannon’s H’ diversity among sample types, with p-values (p), and Holm adjusted p-values (Holm) reported. Pairwise comparisons of microbial community similarity (PERMANOVA, beta-diversity) between sample types, with p-values (p) and false detection rate (FDR) adjusted p-values reported. A “1” following sample type refers to the output from the first sequencing run, a “2” refers to output from the second sequencing run.

|  | Richness | | Evenness | | Diversity | | PERMANOVA | |
| --- | --- | --- | --- | --- | --- | --- | --- | --- |
| Pairwise Comparison | *p* | *Holm* | *p* | *Holm* | *p* | *Holm* | *p* | *FDR* |
| Amphipod 1 x Amphipod 2 | 0.914 | 0.914 | 0.291 | 1.000 | 0.755 | 1.000 | 0.809 | 0.842 |
| Branchial sac 1 x Branchial sac 2 | 0.808 | 1.000 | 0.251 | 1.000 | 0.487 | 1.000 | 0.882 | 0.882 |
| Tunic 1 x Tunic 2 | 0.382 | 1.000 | 0.320 | 1.000 | 0.296 | 1.000 | 0.812 | 0.842 |
| Seawater 1 x Seawater 2 | 0.621 | 1.000 | 0.822 | 1.000 | 0.928 | 0.928 | 0.500 | 0.560 |

**Table S3** Mothur (v.1.43.0) bioinformatics pipeline for processing of raw sequences from Zymo Research Corporation.

| Command | Input File Type | Settings |
| --- | --- | --- |
| make.file | .gz | none |
| make.contigs | .fastq | trimoverlap=T, oligos=515f-806r.oligos, pdiffs=4, processors=8 |
| summary.seqs | .fasta | none |
| screen.seqs | .fasta, .groups | maxambig=0, maxlength=300, minlength=200, maxhomop=8 |
| summary.seqs | .fasta | none |
| unique.seqs | .fasta | none |
| align.seqs | .fasta | reference=silva.nr_v132.V4.align, processors=4 |
| summary.seqs | .fasta, .names | none |
| screen.seqs | .fasta, .groups, .names | start=1967, end=11549 |
| summary.seqs | .fasta, .names | none |
| filter.seqs | .fasta | vertical=T, trump=. |
| summary.seqs | .fasta, .names | none |
| pre.cluster | .fasta, .names, .groups | diffs=2 |
| summary.seqs | .fasta, .count_table | none |
| chimera.uchime | .fasta, .count_table | dereplicate=t, reference=self |
| remove.seqs | .fasta, .count_table, .accnos | none |
| summary.seqs | .fasta, .count_table | none |
| classify.seqs | .fasta, .count_table | reference= silva.nr_v132.V4.align, taxonomy=silva.nr_v132.tax, cutoff=60 |
| remove.lineage | .fasta, .count_table, .taxonomy | taxon=Chloroplast-Mitochondria-Eukaryota-unknown |
| summary.seqs | .fasta, .count_table | none |
| filter.seqs | .fasta | vertical=T, trump=. |
| rename.file | .fasta, .count_table, .taxonomy | none |
| dist.seqs | .fasta | cutoff=0.03, processors=4 |
| cluster | .dist, .count_table | cutoff=0.03 |
| remove.rare | .list, .count_table | nseqs=10, label=0.03 |
| classify.otu | .list, .count_table, .taxonomy | label=0.03 |
| get.oturep | .fasta, .count_table, .list | method=abundance |
| make.shared | .list, .count_table | none |
| count.groups | .count_table | none |
| sub.sample | .list, .count_table | persample=t, size=16572 |
| count.groups | .count_table | none |
| list.otulabels | .list | none |
| get.otulabels | .otulabels, .cons.taxonomy | none |
| make.shared | .list, .count_table | none |

**Table S4** Top 3 NCBI BLASTn results (species, % identity), accession number (Acc. num.), and origin of sequence match for significantly differential OTUs and OTUs of interest between *Amphipoda* sp. and *Ascidia sydneiensis* samples

| OTU | SILVA Identification | BLASTn | Acc. num | Origin |
| --- | --- | --- | --- | --- |
| 00198 | *Ferrimonas* | *Ferrimonas* sp. (100.00%) | MK558695.1 | *Haliclona* sp. (sponge), Kuwait |
|  |  | *Ferrimonas* sp. (100.00%) | MK558692.1 | *Haliclona* sp. (sponge), Kuwait |
|  |  | *Ferrimonas* sp. (100.00%) | MK558665.1 | *Haliclona* sp. (sponge), Kuwait |
| 00256 | *Deltaproteobacteria* | Uncultured deltaproteobacterium (91.24%) | EF123495.1 | *Siderastrea siderea* (infected coral), Bahamas |
|  |  | Uncultured bacterium (89.64%) | KU676558.1 | Surface coastal water, Denmark |
|  |  | Uncultured bacterium (89.64%) | AB534764.1 | Sulfate-reducing enrichment culture, Japan |
| 00062 | SAR116 Clade | Uncultured bacterium (100.00%) | KY275718.1 | Coastal waters, South Korea |
|  |  | Uncultured *Alphaproteobacteria* (100.00%) | KX581267.1 | Arabian Sea open ocean, India |
|  |  | Uncultured *Alphaproteobacteria* (100.00%) | KT731599.1 | Surface seawater, China |
| 00007 | *Anaplasmataceae* | *Anaplasmataceae* bacterium (94.82%) | CP048228.1 | *Eurypanopeus depressus* (crab), USA |
|  |  | Uncultured bacterium (92.03%) | KY282414.1 | Coastal waters, South Korea |
|  |  | Uncultured bacterium (87.90%) | KY253000.1 | *Cambarus chasmodactylus* (crayfish), USA |
| 00037 | *Chitinophagaceae* | Uncultured bacterium (91.70%) | KY522386.1 | Lake water, Canada |
|  |  | Uncultured bacterium (91.30%) | KY521222.1 | Lake water, Canada |
|  |  | Uncultured bacterium (91.30%) | KY520247.1 | Lake water, Canada |
