# Supplementary material for: Distinct microbial communities in an ascidian–crustacean symbiosis
Source: Environ Microbiol Rep. 2024 Feb 21;16(1):e13242. doi: 10.1111/1758-2229.13242 (PMC10881349; doi:10.1111/1758-2229.13242)
Supplement: Supplementary file 2 — Figure S1. Visual comparisons between technical replicates sequenced for this study. OTU richness is plotted for each replicate (A), where samples collected from the same individual of Ascidia sydneiensis have a label starting with A–E, corresponding to the five individuals used in this study. Crustacean samples are noted with an ‘R,’ branchial sac with an ‘S,’ tunic with a ‘T,’ and seawater with a ‘W’. A code followed by a ‘1’ indicates it was the result of the first sequencing procedure, while a ‘2’ indicates the product of the second sequencing procedure. Three samples were only produced in the second sequencing procedure: BR, CT and ET (marked with a black star). An nMDS plot comparing microbiome composition among samples is shown (B), where non‐mathematically derived ellipses indicate sample type: amphipod crustaceans (red), branchial sac (green), tunic (purple) and ambient seawater (blue). A line connects each technical replicate pair. [file EMI4-16-e13242-s002.docx]

**SUPPLEMENTARY MATERIALS**

**Distinct microbial communities in an ascidian-crustacean symbiosis**

Brenna Hutchings^1^, Susanna López-Legentil^1^, Lauren M. Stefaniak^2^, Marie Nydam^3^, Patrick M. Erwin^1^

^1^*Department of Biology & Marine Biology, and Center for Marine Science, University of North Carolina Wilmington, 5600 Marvin K. Moss Lane, Wilmington NC 28409, United States of America*

^2^*Department of Marine Science, Coastal Carolina University, 100 Chanticleer Dr. E., Conway SC 29528, United States of America*

^3^*Department of Biology, SOKA University of America, 1 University Drive, Aliso Viejo CA 92656, United States of America*

**Figure S1** Visual comparisons between technical replicates sequenced for this study. OTU richness is plotted for each replicate (A), where samples collected from the same individual of *Ascidia sydneiensis* have a label starting with A-E, corresponding to the five individuals used in this study. Crustacean samples are noted with an ‘R,’ branchial sac with an ‘S,’ tunic with a ‘T,’ and seawater with a ‘W’. A code followed by a ‘1’ indicates it was the result of the first sequencing procedure, while a ‘2’ indicates the product of the second sequencing procedure. Three samples were only produced in the second sequencing procedure: BR, CT, and ET (marked with a black star). An nMDS plot comparing microbiome composition among samples is shown (B), where non-mathematically derived ellipses indicate sample type: amphipod crustaceans (red), branchial sac (green), tunic (purple), and ambient seawater (blue). A line connects each technical replicate pair.
