# Supplementary material for: Distinct microbial communities in an ascidian–crustacean symbiosis
Source: Environ Microbiol Rep. 2024 Feb 21;16(1):e13242. doi: 10.1111/1758-2229.13242 (PMC10881349; doi:10.1111/1758-2229.13242)
Supplement: Supplementary file 4 — Figure S3. Domain level composition of microbial communities in Amphipoda sp., Ascidia sydneiensis branchial sac, ambient seawater and A. sydneiensis tunic. Horizontal black line (y = 16,572) represents the sequencing depth of all samples. [file EMI4-16-e13242-s003.docx]

**SUPPLEMENTARY MATERIALS**

**Distinct microbial communities in an ascidian-crustacean symbiosis**

Brenna Hutchings^1^, Susanna López-Legentil^1^, Lauren M. Stefaniak^2^, Marie Nydam^3^, Patrick M. Erwin^1^

^1^*Department of Biology & Marine Biology, and Center for Marine Science, University of North Carolina Wilmington, 5600 Marvin K. Moss Lane, Wilmington NC 28409, United States of America*

^2^*Department of Marine Science, Coastal Carolina University, 100 Chanticleer Dr. E., Conway SC 29528, United States of America*

^3^*Department of Biology, SOKA University of America, 1 University Drive, Aliso Viejo CA 92656, United States of America*

**Figure S3** Domain level composition of microbial communities in *Amphipoda* sp., *Ascidia sydneiensis* branchial sac, ambient seawater, and *A. sydneiensis* tunic. Horizontal black line (y=16,572) represents the sequencing depth of all samples.
