# Supplementary material for: Distinct microbial communities in an ascidian–crustacean symbiosis
Source: Environ Microbiol Rep. 2024 Feb 21;16(1):e13242. doi: 10.1111/1758-2229.13242 (PMC10881349; doi:10.1111/1758-2229.13242)
Supplement: Supplementary file 6 — Figure S5. Order‐level composition of microbial communities in Amphipoda sp., Ascidia sydneiensis branchial sac, ambient seawater and A. sydneiensis tunic. Abundance of the 10 most common orders is shown. Horizontal black line (y = 16,572) represents the sequencing depth of all samples. [file EMI4-16-e13242-s001.docx]

**SUPPLEMENTARY MATERIALS**

**Distinct microbial communities in an ascidian-crustacean symbiosis**

Brenna Hutchings^1^, Susanna López-Legentil^1^, Lauren M. Stefaniak^2^, Marie Nydam^3^, Patrick M. Erwin^1^

^1^*Department of Biology & Marine Biology, and Center for Marine Science, University of North Carolina Wilmington, 5600 Marvin K. Moss Lane, Wilmington NC 28409, United States of America*

^2^*Department of Marine Science, Coastal Carolina University, 100 Chanticleer Dr. E., Conway SC 29528, United States of America*

^3^*Department of Biology, SOKA University of America, 1 University Drive, Aliso Viejo CA 92656, United States of America*

**Figure S5** Order level composition of microbial communities in *Amphipoda* sp., *Ascidia sydneiensis* branchial sac, ambient seawater, and *A. sydneiensis* tunic. Abundance of the ten most common orders is shown. Horizontal black line (y=16,572) represents the sequencing depth of all samples.
